# Systems for intricate patterning of the vertebrate anatomy

**DOI:** 10.1098/rsta.2020.0270

**Published:** 2021-12-27

**Authors:** Kevin J. Painter, Mariya Ptashnyk, Denis J. Headon

**Affiliations:** ^1^ Dipartimento Interateneo di Scienze, Progetto e Politiche del Territorio, Politecnico di Torino, Torino, Italy; ^2^ School of Mathematical and Computer Sciences and Maxwell Institute, Heriot-Watt University, Edinburgh, UK; ^3^ Roslin Institute, University of Edinburgh, Edinburgh, UK

**Keywords:** Turing pattern, symmetry breaking, pattern formation, hair, feather, skin

## Abstract

Periodic patterns form intricate arrays in the vertebrate anatomy, notably the hair and feather follicles of the skin, but also internally the villi of the gut and the many branches of the lung, kidney, mammary and salivary glands. These tissues are composite structures, being composed of adjoined epithelium and mesenchyme, and the patterns that arise within them require interaction between these two tissue layers. In embryonic development, cells change both their distribution and state in a periodic manner, defining the size and relative positions of these specialized structures. Their placement is determined by simple spacing mechanisms, with substantial evidence pointing to a variety of local enhancement/lateral inhibition systems underlying the breaking of symmetry. The nature of the cellular processes involved, however, has been less clear. While much attention has focused on intercellular soluble signals, such as protein growth factors, experimental evidence has grown for contributions of cell movement or mechanical forces to symmetry breaking. In the mesenchyme, unlike the epithelium, cells may move freely and can self-organize into aggregates by chemotaxis, or through generation and response to mechanical strain on their surrounding matrix. Different modes of self-organization may coexist, either coordinated into a single system or with hierarchical relationships. Consideration of a broad range of distinct biological processes is required to advance understanding of biological pattern formation.

This article is part of the theme issue 'Recent progress and open frontiers in Turing's theory of morphogenesis'.

## 1. Composite organ structure and development

**Figure 1.**
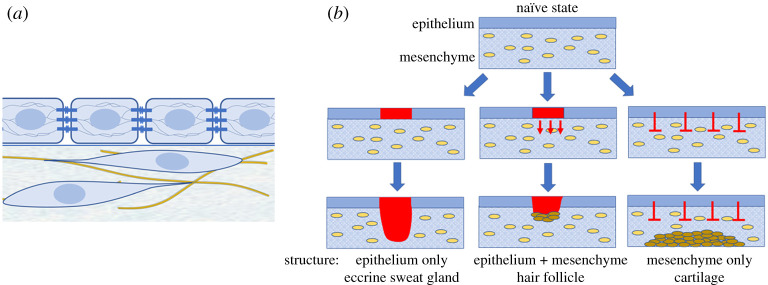
(*a*) A depiction of cellular organization in composite tissues. An epithelium composed of cells attached to one another by junctions sits above a mesenchyme in which cells interact with and move through a fibrous matrix. (*b*) Examples of epithelial–mesenchymal interactions leading to formation of structures composed only of epithelium (a sweat gland), of epithelial and mesenchymal cells (a hair follicle) and only of mesenchyme (a precartilaginous condensation in the digital skeleton) when epithelium inhibits mesenchymal condensation in its vicinity. (Online version in colour.)

In the vertebrate body, many large organs are subdivided into numerous smaller elements. Examples are the hair follicles of the skin, villi of the gut and the extensive branches of the lung, mammary gland or kidney. These periodic entities provide greater surface area and contact interface to enhance absorption, secretion or bidirectional exchange, or in the case of hairs and feathers to trap air for insulation. The organs themselves share a common tissue structure, a composite of a sheet of cells called an epithelium and a connective tissue support, the latter populated with a range of cell types, blood vessels and nerves. The connective tissue is largely constructed by fibroblasts, a cell type which produces extracellular matrix (ECM, such as fibrous collagen and gel-like hyaluronic acid) and which makes up the bulk of the cells during development. These mesenchymal cells have few connections to one another and instead are embedded in a matrix of gel and fibres that they produce and through which they move and pull upon (figure 1). The epithelium is characterized by the attachment of cells to one another through junctions, these junctions also being attached to the fibrous internal cytoskeleton of these cells. This gives the tissue its sheet-like structure and restrains individual cell movement. There is little connective tissue within the epithelium, though between the epithelium and the mesenchyme there lies a thin sheet of specialized ECM called the basement membrane. The cells on the basal layer of the epithelium adhere to the basement membrane, as well as to one another. Epithelia and mesenchyme usually have distinct embryonic origins. With some exceptions, the epithelium largely derives from the ectoderm for structures on or close to the outside of the body and from endoderm for those on the inside. The mesenchyme originates in either the mesodermal layer of the early embryo or in some locations from a later emerging population called the neural crest [1].

The periodic structures that arise and function within these large organs initiate their development prenatally. Their locations first become apparent as patches of tissue with altered cell state, generally defined by the expression of a suite of genes that distinguishes them from their surrounding tissue, and by rearrangements of cell shape and position. Their formation relies on the presence of both epithelium and mesenchyme in close apposition, evidenced by a range of embryological experiments in which these tissues were separated or recombined, with different outcomes depending on location [2]. The periodic structures can have cell contributions from both layers, such as the hair follicle, in which a tightening of epithelial cells to form a placode, and an aggregate of mesenchymal cells to form a condensate, occurs at an early development stage. Other structures can be formed from epithelium alone (sweat gland) [3,4] or a mesenchymal aggregation only (cartilage elements in the limb) [5] (figure 1). Despite their different organ of residence and cellular composition, a commonality in their formation is underlined by evidence that individual genes when mutated coordinately impair the development of many of these structures [6].

Communication between epithelial and mesenchymal tissue layers is thought to be mediated in large part by soluble signals. In particular, gene-encoded proteins that diffuse from their cell of synthesis to bind to highly selective receptors on different cells, triggering a cascade of transduction that alters the receiving cell's state (activity of specific genes in the cell nucleus and altered production of specific proteins) or behaviour (such as the direction of cell movement or prompting cell division), have received a great deal of attention. Classic embryological tissue recombination experiments distinguished these epithelial–mesenchymal influences on one another as being permissive, that is, merely requiring the presence of the counterpart tissue to proceed with pattern formation, or instructive, in which one tissue specifies the spatial pattern itself in the other, acting as a template. Since then, molecular biology approaches have revealed genes specifically associated with cell state changes, these typically emerging prior to morphological or cellular distinctions that identify pattern elements. Also identified have been many of the presumed soluble signals that mediate communication between tissue layers, including a number of different protein ‘growth factors’ and the other proteins required to receive and respond to these signals that travel between cells, whether by simple diffusion or through cellular projections or active cellular processes. These growth factors fall into several different classes, each of which functions in many different organs and stages of embryonic development [7].

## 2. Theories to explain periodic pattern generation

Prior to pattern formation, it appears that many, perhaps all, cells are equivalent in their capacity to form either villi, hair follicles or other periodic elements appropriate to organ type. At this stage, organ rudiments typically have a simple structure, being an epithelial sheet on top of a mesenchyme. Pattern emerges as naïve tissue breaks its initial planar symmetry to yield different physical arrangements and cell states. Cell states are largely a function of differential gene expression; these events are ultimately being controlled by a class of intracellular proteins called transcription factors. The transcription factors operate in gene regulatory networks, giving cell fate determination a deterministic behaviour, often referred to as a programme [8].

**Figure 2.**
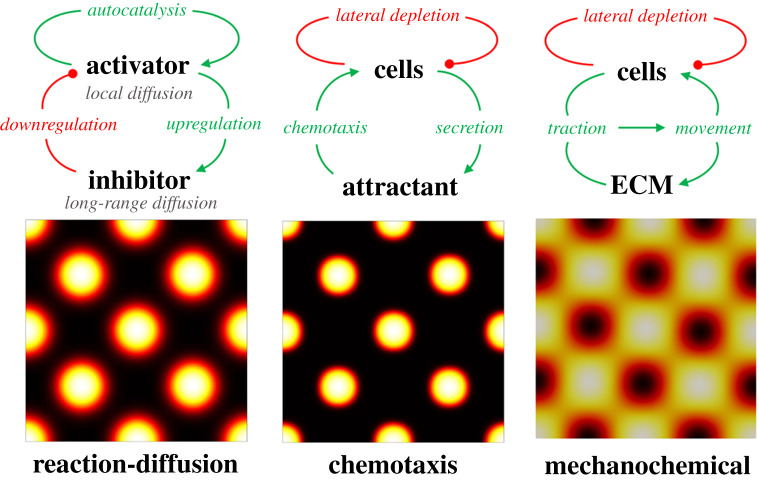
Top row, a schematic with the principal components and interactions for Turing, chemotaxis and mechanochemical models for pattern formation. All are fundamentally based on the principle of local activation and lateral inhibition (LALI), but achieve this condition in different ways. Turing instability analysis on these models reveals a shared capacity to induce periodic patterning with some characteristic wavelength, one determined by the competition between local enhancement (autocatalysis, autotaxis and traction/movement) and long-range inhibition (freely diffusing inhibitors or chemotaxis and mechanical forces capable of drawing in the population, depleting the population over multiple cell lengths). Bottom row, numerical demonstration of representative periodic patterns formed from the models, whether of (Turing) activator concentration or (chemotaxis and mechanochemical) cell density. For each model, we consider a square region of size l×l. (Turing) Equation (2.1) with $f(u,v)=p_{a}u^{2}/(\left(1+k_{1}u^{2})(1+k_{2}v\right))-d_{1}u$, $g(u,v)=c_{i}+p_{i}u^{2}/(1+k_{3}u^{2})-d_{2}v$, where $p_{a}=10,p_{i}=d_{1}=d_{2}=1,k_{1}=k_{3}=0.01,k_{2}=2.2,c_{i}=2.7$, $D_{u}=2.5\times 10^{-4}$, $D_{v}=50D_{u}$ and l=0.75. (Chemotaxis) Equation (2.2) with $\chi (c,a)=\alpha e^{-\beta c}$, f(c,a)=0, g(c,a)=c−a, where $\alpha =0.125,\beta =0.5,D_{c}=D_{a}=0.05$ and l=10. Both Turing and chemotaxis systems are solved with zero-flux boundary conditions and the numerical scheme uses a standard finite volume discretization in conservative form, e.g. see [12]. (Mechanochemical) Under a small strain assumption, the mechanochemical model (2.3)–(2.5) can be simplified into a fourth-order problem for θ; we refer to [13]. Simulations of this problem employ nondimensional parameters $\mu =1=(\mu_{1}+\mu_{2})(1-2\nu )/(E(1-\nu )T),\tau =3.5035=\tau_{u}c_{0}\rho_{0}(1-2\nu )/(E(1-\nu ))$, $\gamma =1=\gamma_{c}c_{0}^{2},\beta =3/112=\beta_{c}/L^{2},\kappa =6.7=\kappa_{u}\rho_{0}L^{2}$ and l=2π, with zero-flux and stress-free boundary conditions. The numerical scheme employed a mixed finite-element method built in FEniCS [14]. (Online version in colour.)

Self-organizing models for morphogenesis suggest that the stochastic differences between cells of an initially essentially homogeneous tissue can be sufficient for the tissue to break its symmetry, i.e. the pattern forms *de novo* and there is no inherent need for a ‘pre-pattern’. Various such models have been proposed, with Turing, chemotaxis and mechanochemical systems receiving perhaps the most attention, see box 1 and figure 2.

Box 1.
**Mathematical models for periodic pattern formation.**

Turing, chemotaxis and mechanochemical models have received considerable attention as mechanisms for morphogenesis. Constituted from different components (such as cells, soluble molecules and extracellular matrix (ECM)) and distinct biophysical processes (such as reaction, diffusion, taxis and forces), they share the capacity to induce periodic patterning through a Turing instability, where a spatially homogeneous solution is destabilized in the presence of some noise.

Turing models rely on the interactions between chemicals that react and diffuse. Simply, a model can be formulated as partial differential equations for two morphogen components, *u* and *v*,

2.1 $$\begin{array}{r} \begin{array}{r} \partial_{t}u=D_{u}\nabla^{2}u+f(u,v)\\ \partial_{t}v=D_{v}\nabla^{2}v+g(u,v). \end{array}\} \end{array}$$

Coefficients $D_{u}$ and $D_{v}$ determine the spatial ranges for diffusion while terms f(u,v) and g(u,v) dictate the molecular reactions. Notably, only certain interactions lead to self-organization, a famous example being ‘short-range activation, long-range inhibition’ [9]. For systems with more than two components, self-organization can occur under a less restrictive set of constraints.

Chemotaxis models rely on the interaction between motile cell populations and their chemoattractants and/or repellents. The pioneering such model for self-organization was proposed by Keller & Segel [10], inspired by autoaggregation behaviour in *Dictyostelium discoideum*. A simple formulation includes a homogeneous cell population, *c*, and its attractant, *a*, governed by

2.2 $$\begin{array}{r} \partial_{t}c=D_{c}\nabla^{2}c-\nabla \cdot (\chi (c,a)c\nabla a)+f(c,a)\\ \partial_{t}a=D_{a}\nabla^{2}a+g(c,a). \end{array}\}$$

Chemotaxis enters the cell density equation via a transport term that (when χ(c,a)>0) describes directed movement of the cell population towards higher concentrations of *a*, i.e. positive chemotaxis. The function χ(c,a) is commonly called the chemotactic sensitivity, measuring the chemotaxis response. Self-organization occurs when positive chemotaxis is coupled to reaction terms including cellular production of the attractant, an ‘autotaxis' that coalesces cells and depletes the population in the surrounding zone in the process.

Mechanochemical models [11] form an even broader class, potentially including variables for cells, chemicals and the ECM. These models are distinguished through their accounting for the forces generated between cells (motile or not) and the tissue within which they sit, leading to more complicated models than those above. Nevertheless ‘minimal’ formulations show that the traction generated by a non-motile cell population can be sufficient to induce self-organization. Here, interactions between the cells and ECM lead to tissue deformation, which in turn transports both the cells and the ECM and leads to clustering. Formulated mathematically, one considers the dynamics of cells (density *c*), and the ECM (density ρ), and supposes their movement is determined by the ECM displacement, *u*, according to standard conservation laws:

2.3 $$\begin{array}{r} \partial_{t}\rho +\nabla \cdot (\rho \partial_{t}u)=0\\ \partial_{t}c+\nabla \cdot (c\partial_{t}u)=0. \end{array}\}$$

The ECM displacement equation is based on principles of linear viscoelasticity

2.4 $$\nabla \cdot (\sigma (u)+\tau_{u}\phi (c,\rho )I)=\kappa_{u}\rho_{0}u,$$

where stress is generated by viscoelastic deformations of ECM and cell traction forces,

2.5 $$\begin{array}{r} \sigma (u)=\mu_{1}\partial_{t}e(u)+\mu_{2}\partial_{t}\theta I+\frac{E}{1+\nu }\left[e(u)+\frac{\nu }{1-2\nu }\theta I\right],\\ \phi (c,\rho )=\frac{c}{1+\gamma_{c}c^{2}}(\rho +\beta_{c}\Delta \rho ). \end{array}\}$$

In the above, $e(u)=(\nabla u+\nabla u^{T})/2$ denotes the symmetric gradient of the displacement *u* and θ=∇⋅u, the Young modulus and Poisson ratio are denoted by *E* and ν, respectively, and $\mu_{1}$ and $\mu_{2}$ are viscosity constants. Further, $\tau_{u}\phi$ is the traction force exerted by the cells, where $\tau_{u}$ is the measure of the traction force generated by a cell, $\rho_{0}$ is the initial ECM density, *I* is the identity tensor, $\kappa_{u}$ is an elastic parameter characterizing the substrate attachments, $\beta_{c}$ is a measure of the long-range cell-ECM interactions, $\gamma_{c}c^{2}$ models contact inhibition as the cell density increases and $\gamma_{c}$ is a measure of how the force is reduced due to neighbouring cells.

The Turing system is the best known and, as the pioneer, the most influential model for periodic patterning in biological systems [15]. Turing's remarkable insight was that the twin actions of chemical diffusion and reaction could be sufficient to amplify the stochastic heterogeneities within an initially near-homogeneous distribution into a pattern. The pattern would presumably be translated into an alteration of tissue morphology (meaning shape or structure), hence Turing dubbed these hypothetical chemicals ‘morphogens’. At its simplest, and most intuitive, it requires just two morphogens: an activator that promotes its own production and an inhibitor, the production of which is likewise driven by the activator but acts to suppress the activator. When the inhibitor has a greater range of action than the activator a periodic pattern of activator and inhibitor concentrations can emerge. This idealization yields more complex systems composed of many more molecular components, though these can often behave in a similar manner to the presumed simple two-component systems [16,17].

Turing systems rely on the processes of reaction (chemical synthesis and decay) and diffusion, and for the purposes of the present paper, we interchangeably use ‘Turing’ or ‘reaction–diffusion’ as self-organizing models built on these principles, i.e. where there is no *explicit* representation of an underlying cell population (mathematically, ‘reaction–diffusion’ describes a far broader class of models). There is, though, an *implicit* assumption of the cellular tissue in these models, as the reception and response mechanism for most diffusible biological chemicals requires the action of many proteins in cells. Thus, at their core, Turing systems may be expected to have a set of at least two regulated factors that vary greatly in their expression over the timecourse and spatial scale of patterning, becoming focalized from a broad initial distribution to define the pattern. A change in any cell's state is then instructed by its concentration of these regulated factors, in the simplest conception triggering cell fate change and activating a gene regulatory network upon attaining some threshold in concentration. However, the many distinct proteins that receive, interpret and respond to these chemical signals may remain produced homogeneously and steadily throughout the patterning field, and are simply required to permit the operation of the system. Removal of either the regulated instructive or the permissive factors can lead to the abolition of symmetry breaking. Most experimental studies have focused on finding the regulated diffusible components of patterning systems and their interactions, rather than on defining each component of the permissive systems that serve as machinery for signal reception and response [18]. These efforts are akin to circumnavigation attempts by plotting out the interacting activator and inhibitor loops, prior to filling in all parts of the molecular map. It is notable that while the concepts of chemical production and diffusion are simple to grasp, measuring these parameters in real biological systems is difficult. One part of this difficulty rests in the two-step process of protein production via an mRNA intermediate, while a second part is down to the technical challenge of detecting extracellular molecules present in small quantities [19]. However, even with limited direct measurement of relevant molecular parameters, experiment—and theory—led approaches are refining the range of parameter space that permits pattern formation by reaction–diffusion systems of varying complexity [20,21].

Other systems for symmetry breaking have been proposed that do not rely on the action of antagonistic chemical signals, but act through a phenomenologically similar combination of positive and negative feedback processes. One set relies on cell and/or tissue movements to generate periodic aggregates or condensations of cells, therefore demanding an explicit equation for the cellular dynamics. In chemotaxis models, initially dispersed but mutually attractive cells clump together by producing a chemoattractant. As small aggregates form they produce more of the attractant, simply as a result of increased cell density, and so more cells are recruited to that location. Aggregation of cells may also impair their mobility as adhesion between them is likely to occur. Mechanical models suggest that cells strain the ECM, with the resulting tissue deformation leading to the displacement of both cells and the matrix, a feedback loop that can also lead to cell clustering. Mechanical influences in tissue patterning carry the advantage that forces can propagate more quickly through a tissue than diffusing chemicals, expanding spatial range and enabling more rapid communication across the domain. These models differ fundamentally from reaction–diffusion systems in that they do not involve a templated stage of a chemical prepattern which drives subsequent cell rearrangements. Rather, cell behaviour and local density are direct reflections of patterning events as they occur. The relevant parameters of cell movement, cell morphology and cell density are readily observable and quantifiable in organ culture systems [22,23], and methods for measuring and manipulating tissue mechanics are advancing [24].

All of these systems can generate a pattern by amplifying the initial noise in a near-uniform system, whether the noise lies in random differences in local chemical concentrations, cell densities or mechanical strain. Turing assumed initial homogeneity out of analytical necessity, recognizing the rarity of this condition within the developing organism (‘*Most of an organism, most of the time, is developing from one pattern into another, rather than from homogeneity into a pattern*’, Turing 1952). In many specific developmental situations the inducing noise or asymmetry may not be random but instead influenced by field inhomogenities, such as a pre-existing anatomical feature. Thus symmetry breaking as a property of a network or system should be viewed as a potential rather than an event, as many systems capable of breaking symmetry and producing pattern *de novo* will in fact be guided by pre-existing anatomical landmarks which bias the system to pattern in a nonrandom manner. That is, while a symmetry breaking system can be defined by its ability to break symmetry *de novo*, in an embryonic context it is seldom likely that a truly symmetric or homogeneous field is the starting point for pattern formation.

Considering these cell behaviours and attributes, the structure of composite epithelial–mesenchymal organs lends itself to the operation of different types of pattern-forming systems. Relatively immobile epithelial cells produce and respond to diffusible signals, thus closely fitting the framework offered by the Turing or reaction–diffusion system. Motile fibroblasts also produce and respond to signals, but reside and move through the ECM, facilitating symmetry breaking through chemotaxis or mechanical means. However, the behaviours of these systems in terms of the patterns that they produce, their relationship to domain growth, and the types of pattern changes that they can undergo, are very similar (figure 2). As such, description of the final state of the pattern may be of marginal use when it comes to determining the mechanisms that produced it [25].

The ability of these different processes to drive pattern formation is supported by spatial patterns produced by some microbial cells when at high density. A Turing system operates in filaments composed of a chain of cyanobacteria. Under conditions of low-nitrogen availability every approximately 10^th^ cell assumes a heterocyst identity in order to fix atmospheric nitrogen for the collective. This occurs without rearrangement of cell positions, and instead through the diffusion of small proteins that inhibit the activator protein HetR system [26]. Conversely, bacterial patterning systems relying on mechanical interactions of moving cells exist. A striking example is a flower-like pattern that emerges from cocultures of *E. coli* and *A. baylyi* bacterial species on soft agar, where the stiffness of the agar substrate influences the spatial arrangement. Physical interactions between the highly motile *A. baylyi* and the non-motile *E. coli* lead to the expansion of two-dimensional patterns reminiscent of flower growth [27].

The clearest evidence for the operation of local activating long-range inhibitory systems in the generation of anatomical patterns in vertebrate organs comes from the behaviour of patterns in perturbed conditions. In the embryonic skin, the hair and feather patterns respond to boundaries, field growth and alterations in cell–cell signalling in ways easily reproduced by local activation-long range inhibition processes. These have been idealized as simple activator-inhibitor type reaction–diffusion systems [28], and subsequent work has extended, and also challenged, the degree to which they conform to such idealizations. Here, we will focus on recent insights into the roles of cell signalling, cell movement and mechanical influences on periodic patterning of avian and mammalian skin to highlight issues related to assessing the relative importance of different processes to biological pattern formation. We seek to draw out the key concepts underlying the operation of these systems, but do not attempt a full review of the literature on pattern formation, several excellent examples of which have been published recently [18,29,30].

## 3. Patterning of hair follicles: chemotaxis subordinated to reaction–diffusion

**Figure 3.**
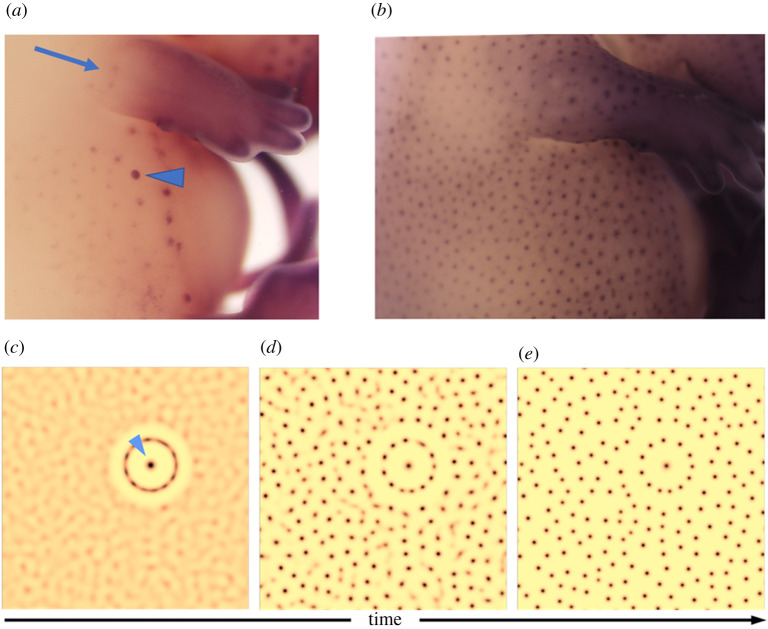
Initiation of hair follicle formation in mice. Embryonic day 13.5 embryo (*a*) shows hair follicle primordia (dots) initiating around and spreading away from the mammary gland primordium, indicated by arrowhead. The mammary primordium inhibits hair follicle formation in its vicinity. Spontaneous patterning sites also emerge (arrow). By embryonic day 14.5 (*b*), the skin is populated by a periodic array of hair follicle primordia. Stained for transcript encoding β-catenin by the *in situ* hybridization method described in [22]. (*c*,*d*,*e*) Time excerpts from a simulation of a standard activator-inhibitor system, augmented with a focalized region (arrowhead) of modulated signalling, e.g. representing a mammary gland primordium. This prepattern accelerates the appearance of the surrounding ring of spots, while further away spots emerge *de novo* and in less orderly fashion. Simulations: equation (2.1) are solved with $f(u,v)=\alpha +u^{2}/v-\beta v$ and $g(u,v)=u^{2}-v$, with $\beta =0.5,D_{u}=1.25\times 10^{-4},D_{v}=100D_{u}.$ Here $\alpha =0.4I_{\Omega }$, where $I_{\Omega }$ is the indicator function and Ω represents the circular region of radius 0.01 centred at the end of the arrowhead, representing a fixed but spatially concentrated source of u. We use a square region of size l=5 (with zero-flux boundary conditions) but crop panels to focus on patterning near the source term. (Online version in colour.)

Hair follicles are numerous and form through epithelial–mesenchymal interactions, with both tissue layers contributing cells to the mature structure [31]. Different types of hair follicles arise in sequence across the body of the mouse embryo, the best-studied species. Consistent with their operation in a Turing reaction–diffusion mechanism, some genes are initially expressed uniformly across the early epithelium but become restricted to a punctate pattern that anticipates the sites of hair follicle formation [32]. The earliest follicles to be specified are the whiskers or vibrissae on the face. On the trunk, the first arising primary hair follicles appear in a ring around the mammary placodes, which sets an inhibitory boundary for their initiation, and follicle formation spreads in a wave from there. This can be understood as the inhibitory effect of the mammary gland, which produces hair follicle inhibitors, puncturing the equilibrium of the naïve skin by initiating a boundary to nucleate pattern formation, as shown in simulation (figure 3). However, this initiation site is not essential to trigger pattern formation: other unconnected body sites can undergo spontaneous follicle formation and skin collected from the embryo and cultured in the absence of mammary glands (or indeed any small piece of embryonic skin) will readily produce a hair follicle pattern in culture. New hair follicles emerge between the existing ones, so that the vast majority of follicles have their location defined by pre-existing cues [33]. The prospective hair follicle locations are identifiable morphologically as epithelial placodes underlain by mesenchymal condensates (figure 1). The placode and the condensate form through cell recruitment, then the placode cells divide quickly, though the condensate cells do not, and the epithelial component grows down to form a cylinder that will become the hair follicle. New hair follicles continue to form for about a week in mice, with the embryos growing significantly in this period.

Glover *et al.* [22] assessed the relative timing of cell rearrangement and cell state changes during primary hair follicle patterning, finding that patterned change in cell state in the epithelium precedes the accumulation of cells in the underlying mesenchyme. Thus, a prepattern of cell states precedes observable morphological changes, hinting at their setting through a reaction–diffusion mechanism. Here, in an attempt to define the molecular interactions underlying patterning, the authors used mRNA half-life as a filter to identify those mRNAs with a decay rate sufficiently rapid to allow turnover on the timescale of pattern formation—approximately 10 h. Focusing then on the Wingless-Int (WNT), bone morphogenetic protein (BMP) and fibroblast growth factor (FGF) intercellular signalling pathways, they defined a set of interactions between extracellular components that is considerably more complex than the classical 2-component idealization. This network contains no direct positive feedback and instead is dominated by negative feedback. Nevertheless, analysis reveals that the structure of this network is capable of breaking symmetry. It may, however, be incomplete, or may have subelements that play small roles in pattern formation. In general, the field of biological pattern formation and specifically that of Turing systems faces a challenge in assessing the completeness of proposed models, and that of identifying when there are missing or possibly redundant patterning factors. Deeper integration between experimental and computational approaches will be essential, for example, via a cycle in which simulations are used to determine a candidate set of network topologies capable of generating a given gene expression pattern, with experiments pruning the set in turn [21].

By altering the conditions in which skin tissue is maintained in culture, Glover *et al.* [22] revealed the potential for autonomous self-organization in the mesenchyme, one that is normally suppressed and subordinated to the largely epithelium-led reaction–diffusion system. A universal augmentation of FGF combined with suppression of BMP permitted aggregation of the mesenchymal cells into periodic condensates, without any apparent involvement of the epithelium. This mesenchyme-only pattern was slower to form than that generated when epithelial reaction–diffusion patterning operated.

Investigation of the mesenchyme-only patterning found that this process is far more sensitive to modulation of another intercellular signalling pathway, transforming growth factor β (TGF-β) than when the system had been unperturbed. Either suppression or universal augmentation of TGF-β signalling resulted in failure of mesenchyme-only patterning, while having only modest effects on hair follicle patterning under normal conditions. TGF-β is produced by mesenchymal cells and has two observable effects on them. First, when widely available, TGF-β promotes aggregation of mesenchymal cells at sources of FGF. Second, TGF-β itself is a chemoattractant for these cells, making them, in the absence of local FGF as a guiding influence, mutually attractive. Thus, a field of mesenchymal cells will, upon attaining an appropriate density, spontaneously initiate local clustering events serving to nucleate aggregates. This locally increased density further amplifies the chemoattractant at that location, drawing in more distant cells and thereby depleting cells from the region surround the incipient aggregates. This can be viewed as a form of lateral inhibition echoing that found in reaction–diffusion mechanisms that operate through the depletion of a limiting substrate or ligand rather than active inhibition. Mesenchymal cell depletion by condensations is, however, barely apparent during normal hair follicle patterning; only a small fraction of the total mesenchymal cell population is recruited to form condensates, under local direction from the placode produced FGF20 and SHH (Sonic Hedgehog), and perhaps other factors. Indeed, the *Fgf20* mutant mouse illustrates the independence of the epithelial patterning system from that of the mesenchyme. *Fgf20* loss of function permits periodic patterning in the epithelium only, broader and more striped than normal, but clearly periodic, without any apparent mesenchymal contribution to the pattern [34].

Thus, primary hair follicle formation is driven by an epithelial reaction–diffusion system, its signalling components produced within the largely immobile epithelial cells to create a prepattern template. This system is connected to a mesenchymal patterning system through local production of FGF20, and by high levels of BMP signalling that suppress autonomous mesenchymal aggregation. Mesenchymal cells are motile and attracted to sources of FGF20, which are laid out in a template generated by the reaction–diffusion system. The loss of *Fgf20* breaks this link between the tissue layers, permitting epithelial patterning and placode formation, without engaging the mesenchyme [34]. By changing the signalling environment to one in which BMP is low and FGF high, the epithelial signalling system is suppressed, probably by FGF alone, and the motile mesenchymal cells aggregate through TGF-β driven self-attraction. Hence, in mouse skin, a chemotaxis-based patterning mechanism within the mesenchyme is subordinated to a reaction–diffusion system that operates largely in the epithelium. Appropriate conditions permit the separation of these two patterning potentials, permitting epithelium-only patterning through reaction–diffusion or mesenchyme-only patterning through a chemotaxis mechanism [22].

## 4. Origin of the patterning field and initiation of symmetry breaking

Both cell signalling and cell movement systems raise the question of how the patterning field itself develops to the point at which symmetry breaking can occur, a question little addressed in the theoretical literature. Common practice has been to initiate a theoretical model with the rate constants, diffusion coefficients or other parameters fixed at a value likely to yield the desired outcome, e.g. a spotted pattern with wavelength characteristic of feather/follicle spacing. It is also possible to determine the polynomial form of reactions in the reaction–diffusion system that would generate the desired pattern for a given set of parameters, e.g. rate constants, diffusion coefficients [20]. Such approaches are natural from the perspective of comparing theoretical patterns with the visible pattern, but circumvent the question of how the tissue reaches that point to begin with: why does the pattern emerge when it does, and not later, or earlier? The implicit assumption is that all molecular components are in place and their interactions all switched so as to drive the emergence of pattern. However, this ignores the developmental origin of the patterning field itself and its initial properties.

**Figure 4.**
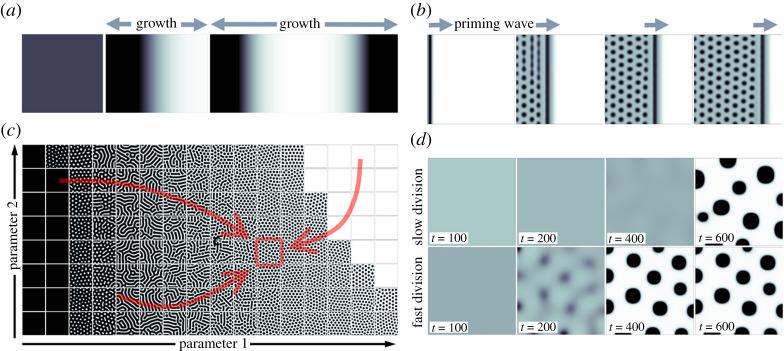
Pattern initiation. (*a*) Physical domain growth is a natural trigger, but will typically initially give rise to one or two pattern elements (indicated as dark areas). (*b*) Spread of a ‘priming wave’, here upregulating expression/production of an activator component, causes an orderly shift of the tissue into a patterning-permissive state. (*c*) A typical Turing parameter space will generate various pattern forms. Tuning components/interactions to reach a desired, or evolutionarily selected, pattern outcome could involve various routes through the parameter space. (*d*) Simulations of chemotaxis models for a population initialized at a low density and growing with distinct growth rates. The slowly dividing population (top row) clearly shows a delayed onset of patterning, even if the final pattern form is qualitatively equivalent. (*a*–*c*) equation (2.1), with f(u,v) and g(u,v) and parameters as specified for figure 2, except where stated otherwise. For (*a*), the equations have been augmented to include a uniform growth along with the direction of the horizontal axis, such that over the three panels shown the domain grows in size from 0.05×0.05 to 0.12×0.05. For (*b*), the equations have been augmented to include a wave that moves from left to right, which acts to decrease parameter $c_{i}$ from 5.4 to 2.7. For (*c*), we plot the pattern that forms for different parameter pairs (Parameter 1, Parameter 2), where Parameter 1 is $c_{i}$ and Parameter 2 is $k_{2}$. Other parameters are held fixed. (*d*) Equation (2.2), formulated and parametrized as in figure 2, except now $f(c,a)=re^{-rt}$. This describes an influx of cells (of equivalent eventual total population), where for the top row r=0.01 and for the bottom row r=0.05. (Online version in colour.)

One possibility is that all is primed to pattern, but patterning is triggered through the growth of the ‘physical field’, i.e. a field initially small relative to the pattern wavelength triggers symmetry breaking upon growth to a certain size [35] (figure 4*a*). However, growth in this way will initially generate only one or two pattern elements, whereas embryonic skin has ample space for numerous hair or feather follicles several days before they first emerge. Physical field size, here at least, is not likely to be the determining factor for initiating pattern formation.

A second possibility, therefore, is that there is a steady extension in the size of the ‘primed field’, for example, a spreading wave that introduces a key signalling component, lifting the patterning system into the self-organization parameter space (figure 4*b*). What, though, needs switching on or off? Components in a signalling network must be activated in expression, following an upward trajectory of expression of the relevant gene. Gene expression is not smooth but rather defined by bursts of activity in any individual cell; this episodic production potentially increases noise between cells on initiation of gene expression. Alternatively, it is conceivable that all molecular components required to form a pattern are present, but that a pattern-inhibitory factor inhibits their interactions, with this factor steadily diminishing in a way that permits the initiation of patterning. Degradation of macromolecules is likely to be more continuous than is mRNA synthesis, reducing the noise arising from the operation of the reaction. In avian skin, an element of this blockade of pattern formation by an inhibitory factor can be seen when BMP signalling is elevated, interacting with retinoic acid produced on the neck to suppress feather patterning in that region of the skin [36].

A further mechanism that would allow a field to grow and establish itself prior to any symmetry breaking could be through suppressing the noise required for the initiation of pattern formation. Such suppression could, in theory, be achieved through remarkably precise production rates of relevant molecules across time and space in individual cells, though this seems unlikely based on our knowledge of cell-to-cell variability and the mechanisms of gene expression. However, since neighbouring cells are unlikely to be in phase regarding their levels of noise, the averaging of state across neighbouring cells may serve to suppress noise. Active mechanisms that dampen noise and impose partial robustness on a naïve and unpatterned state are highly plausible, preventing local deviations from homogeneity forming and initiating pattern formation. Such noise dampening could be contributed to by rapid negative feedback as a form of autosuppression, an extremely common phenomenon in biological pathways [37].

Reaching a desirable pattern form and wavelength, therefore, is likely to involve smooth or jagged passage through the system's parameter space (figure 4*c*). Is the same final outcome expected from any given route? What control does this permit for patterning in a robust spatio-temporal sequence? Theoretical modelling of this tissue transition from a prepatterned to patterned state could clearly yield some valuable insights.

Chemotaxis- or mechanical-based patterning mechanisms are somewhat easier to study than reaction–diffusion systems, as cells are more readily and accurately observed experimentally than molecules. In particular, labelling of cells through the production of fluorescent proteins permits observation of their location, shape and dynamic behaviour under time-lapse microscopy [22,23]. The initiation of patterning for a chemotaxis system in particular will be highly dependent on the concentration of chemoattractant that cells produce, their responsiveness to the chemoattractant, and on cell density, such that below a threshold density of dispersed cells the rate of production and decay of the chemoattractant will cause no pattern formation. Above that threshold rapid aggregation-driven patterning may occur. Importantly, this difference in behaviour need not be accompanied by any difference in the state or behaviour of individual cells, but be triggered by cell density changes only (figure 4*d*). Thus, a steadily building density of dispersed cells can become unstable through chemotaxis when a threshold density is attained and spontaneously pattern into spatially periodic aggregates. A mechanical model is similar in this regard, with a gradually increasing density leading to increased deformation of the ECM and eventually inducing aggregation.

## 5. Feather pattern formation: integration of patterning systems

A mesenchymal cell density dependence for initiating pattern formation has long been recognized in feather formation. Feathers are produced only in regions of the body with high embryonic mesenchymal cell density; the low-density regions remaining unfeathered [38]. The accumulation of mesenchymal cells at sites of feather primordia, which are much larger than those of hair follicles, has been suggested to play a key role in symmetry breaking in chicken skin [39]. However, the characteristics and behaviours of individual cells are not different between high- and low-density mesenchyme [23], and while harvested mesenchymal cells reconstituted at low density do not permit feather pattern formation, the same cell population reconstituted at high density does [40]. Thus, the state or identity of skin mesenchymal cells seems to be of less importance than their density, in the context of permissiveness for feather patterning.

**Figure 5.**
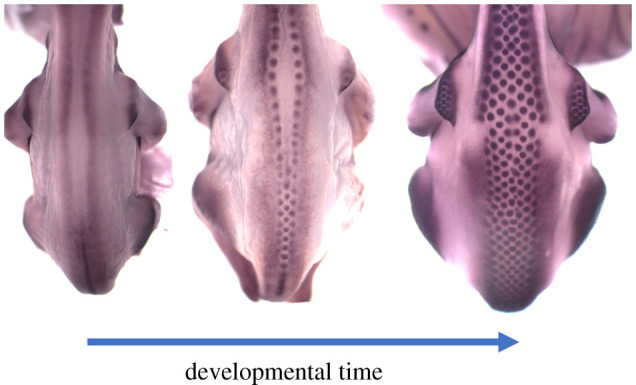
The progression of feather pattern formation in chicken embryos from embryonic day 6.5 to day 7.5. Detection of the transcript encoding β-catenin by *in situ* hybridization as described in [23] reveals the regions competent to undergo pattern formation and the feather primordia. Competence to initiate symmetry breaking is not widespread, but initially restricted to a forked strip running up the back. As the waves of feather formation spread, the existing primordia guide the positioning of those in each successive row. (Online version in colour.)

Feather formation occurs in a wave that spreads outwards from the skin overlying, or running along either side of, the spine (figure 5). This wave of feather primordium generation is preceded by both laterally expanding waves of increasing cell density [23,41] and gene activation [23]. In addition, a number of signalling molecules are expressed and active in feather buds, largely held in common with hair follicles and with approximately the same roles and relationships: FGFs attract mesenchymal cells [23,42], WNTs are required for feather tract and bud formation [43] and BMPs are inhibitory [23,44].

Much of the effort to understand the basis of feather development has focused on the mesenchyme as a driver of pattern formation. Bailleul *et al.* [41] studied proliferation and the resulting cell density changes as the primary trigger of feather pattern formation, identifying characteristic differences in the initial placement of dense cellular regions in different species of birds. In each species, feather patterning is initiated in and follows the expanding regions of high cell density. Supporting the importance of cell density, suppressing proliferation in skin explants reduced both cell density and the spread of the feather forming wave. The localized initiation of periodic pattern formation in most of the species studied, either as a stripe along the midline or as a pair of stripes on either side, highlights the inhomogeneous nature of the patterning field from its outset.

A mechanical process for symmetry breaking in chicken skin was proposed by Shyer *et al.* [45]. Here, feather patterns arising in embryonic skin cultured on substrates of different stiffness were assessed, finding that the size and periodicity of the feather buds produced varied with different physical conditions. Contraction of the skin, which occurs within tens of minutes once removed from the embryo, is driven by the skin's mesenchymal layer. This process of contraction compresses the overlying epithelium, triggering mechanical activation of the β-catenin protein. β-catenin is predominantly found in association with the junctions connecting epithelial cells, but is displaced from this subcellular location by tissue compression. In other systems, however, β-catenin displacement is driven by the application of stretch to cells [46]. Whatever the nature of the tissue distortion, the displaced β-catenin protein is free to enter the nucleus and alter gene expression, and thereby cell state. This thus represents a well-understood link between a mechanical process and a chemical one, with cell to cell junctions and their many associated proteins serving as mechanosensors [24]. β-catenin is a key mediator of WNT signalling, its activation triggered by a chain of events from extracellular reception of soluble WNT signals to stabilization of the otherwise short-lived cytoplasmic β-catenin. This permits β-catenin accumulation and nuclear entry. Thus, mechanical cues can mimic or potentiate WNT signalling. This model, though, does not address the basis for pattern initiation and spread from the midline, or assess whether there are mechanical distinctions within the skin on the scale of the pattern wavelength itself.

Solely mesenchyme-driven models for the periodic arrangement of placodes fail to account for the role of epithelium in symmetry breaking. The loss of FGF20 function in the chicken abolishes all indications of feather formation, including the loss of mesenchymal condensates, but this factor is produced only in the epithelium [47]. Thus, some epithelial influence is required to guide mesenchymal cell condensation in avian skin. Ho *et al*. [23,48] proposed an integrated epithelial–mesenchymal model for feather patterning, in which a spreading wave of gene expression interacts with the cell density wave to define a wavefront competent to undergo periodic patterning. Production of an extracellular protein, Ectodysplasin A (EDA), expands from the feather tract origin and activates FGF20 production in the epithelium, to a degree. FGF20 attracts mesenchymal cells, the movement of which is presumed to locally compress the overlying epithelium. Tissue compression in turn triggers β-catenin activity, thereby rapidly increasing FGF20 production and generating positive feedback. Mesenchymal cell condensates also activate the production of BMP4, known in similar contexts to be stimulated by compression of mesenchyme [49], a diffusible factor that suppresses FGF20 expression. A periodic pattern forms with islands of high FGF20 and BMP4 production in feather primordia, with the greater range of BMP4 and sequestration of mesenchymal cells into condensates both acting to suppress feather formation in the surrounds. The sequestration of cells in feather condensates differs from cell rearrangement in mice, wherein only a small fraction of mesenchymal cells is recruited to the hair condensates, leaving most of the cells in place and thus permitting the formation of new hair follicles as the skin grows. In chickens, unlike mice, new feather follicles are not inserted into the pattern behind the wave, even with increasing skin area due to embryo growth. Mathematical formulations of this model, including both the initiating waves and the feedback loops that generate a pattern, highlight the critical thresholds placed on cell density and recapitulate the orderly formation of feather tracts [50].

In this model, the wave crossing the skin is composed of increasing density of mesenchyme and changes in epithelial cell state (particularly the expression of EDA). However, EDA-triggered epithelial FGF20 production can only activate a productive positive feedback loop if sufficient mesenchymal cells are nearby to be recruited and to generate an aggregate capable of stimulating further FGF20 production. EDA thus interacts with cell density, lowering the density threshold required to produce a feather primordium, but incapable of triggering feather formation when cells are too sparse to be recruited in numbers.

The initiation and operation of this wave means that true symmetry is broken at most once in this unfolding process, producing a periodic arrangement of feather primordia along with the initial longitudinal stripe at the centre of the tract (figure 5). Subsequent to this event, patterning occurs along the wavefront, but the new feather placode positions are determined by the previously laid out row. However, the periodic pattern generator in chicken skin is capable of breaking symmetry across a two-dimensional domain. This was demonstrated by first blocking pattern formation while still allowing expansion of the competence waves of cell density and EDA production and then releasing this block, which resulted in spatial patterning across the broad competent region [23].

**Figure 6.**
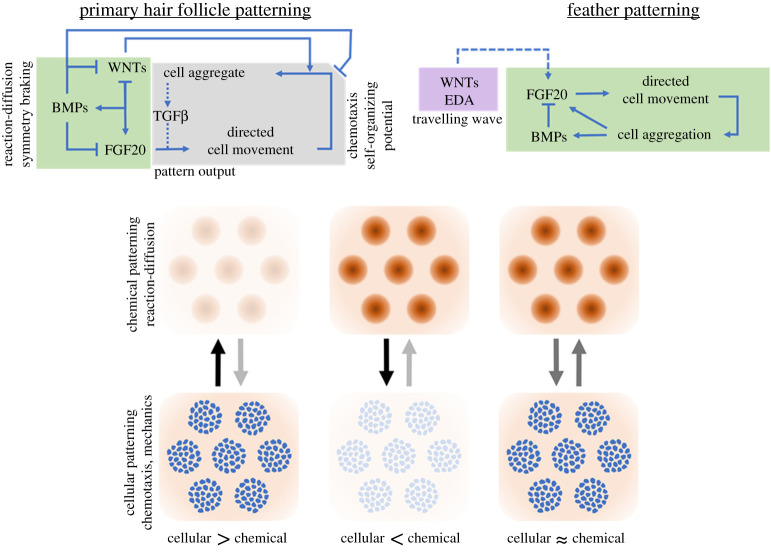
Models for mouse hair follicle formation and feather patterning. In mice, a set of signalling interactions (simplified here) between WNT, FGF and BMP families establishes a prepattern template of foci of epithelial FGF20 production. Mesenchymal cells respond by accumulating at these foci, augmented by TGF β signalling. Under conditions of suppressed BMP and ubiquitous FGF, the mesenchymal cells self-organize through TGF β-guided chemotaxis. Feather pattern formation is permitted by a spreading wave of EDA signalling, initiating FGF20 expression. Mesenchymal cells aggregate at FGF sources, mechanically triggering the production of FGF and BMP, which suppresses FGF20 expression. Below, chemical models, e.g. Turing-like reaction–diffusion, create a periodic molecular template across a uniform density tissue; cellular models, e.g. chemotaxis or mechanochemical models, lead to periodic density structures such as clusters. Entwined, one mechanism could be subordinated to the other, e.g. a ‘chemical > cellular’ relationship where the molecular output directs cell movement through chemoattractant sources. Alternatively, the two mechanisms may have equal import, tightly coupled and substantially impacting one another's dynamics. (Online version in colour.)

Thus, the mechanism for generation of periodic patterns in mouse and chicken skin differs. In mice, the skin breaks symmetry largely through an epithelial reaction–diffusion system, subordinating a cell movement-driven patterning potential in the mesenchyme. Chicken skin instead employs a fully integrated reaction–diffusion–mechanical–chemotactic system (figure 6). The distinction between patterning modes in these species is demonstrated by the effect of FGF20 loss. While FGF20 is required to recruit mesenchymal cells in both species, mouse FGF20 loss permits epithelial periodic patterning with no mesenchymal condensation [34]. In chickens, however, FGF20 absence leads to no pattern whatsoever, in either epithelium or mesenchyme [23,51,52]. This demonstrates the importance of mesenchymal condensation as a process integral to symmetry breaking in chicken skin, but as an output in the patterning of mouse primary hair follicles.

## 6. Outlook

In mouse and chicken skin, we find that the systems underlying periodic patterning appear to be more complex at the molecular level than suggested by the original two-component idealizations of Turing-type models. With multiple components, reaction–diffusion models can be constructed such that knocking out an individual element impacts weakly on patterning, and consequently this complexity may have been evolutionarily selected for to achieve robustness and repeatability of pattern formation, or for tuning of the pattern's characteristics. These added influences may push an originally simple symmetry breaking system towards building a scaffold of genetic influences that ultimately take control of much of the process [53]. In addition, general observations on genetic and molecular processes suggest that accumulation of unnecessary complexity of networks may be tolerated, and likely, as long as the phenotype under selection maintains its characteristics (at the molecular level ‘function diffuses’) [54,55]. This suggests that the clearest insights into the conceptual forms of pattern-forming systems might come from the study of patterns that have evolved recently and where the precise outcome of the pattern is not under strong selection. Such a recent and variable evolutionary innovation may be coat colour markings on cats, which are variable between individuals and species. Here progress in understanding the genetic basis of variant patterns and underlying developmental processes suggests a simple WNT-centred system, operating on a large spatial scale in fetal feline skin [56]. Still simpler and more readily understood systems of interactions may underlie patterns that have never been subjected to natural selection at all, notably experimental artefacts such as the creation of flower pattern from bacterial cocultures [27]. Beyond such examples, research into pattern formation in the vertebrate anatomy should be prepared to tackle complexity and seek suitable levels of analysis and understanding to progress.

Skin morphogenesis provides a system in which evidence is found for both the chemical (reaction–diffusion/Turing) and cell movement (chemotaxis/mechanochemical) paradigms of self-organization. Commonality in structure and gene expression between skin and many other developing tissues naturally raise questions on the significance and ubiquity of this finding. Pattern generators could be placed in different hierarchical relationships (figure 6), interacting in different ways. For instance, the chemical template created by a Turing system could feed into a movement-based model through creating chemoattractant sources, modulating ECM properties or regulating cellular adhesiveness. On the other hand, by aggregating cells into clusters, movement-based models could alter signalling dynamics through compression-induced modulation of activity or simply by locally sequestering a key signalling component. A nuanced understanding of the degree to which dual-patterning systems impact on patterning is undoubtedly challenging, yet perfectly primed for an integrated theoretical-experimental approach.

Beyond the relative simplicity of the skin's planar patterns, understanding the three-dimensional patterning of branched organs represents greater complexity and challenge. Different modes of branching dynamics have been observed using recently developed animal models and general rules describing the branching process derived from these [57]. The identification of molecules and subsystems analogous to Turing-type interactions [58,59] and theory-driven approaches [60], with experimental assessments [61], have generated a framework to understand how branching structures emerge. Unifying branching patterns and the periodic patterns of the skin and other tissues, though, is their emergence from the interactions between epithelial and mesenchymal tissue layers. Probing further into the molecular and mechanical properties of this organ constitution will be critical for understanding its adaptability and capacity to drive the emergence of form in the vertebrate anatomy.

## Data accessibility

This article has no additional data.
